# Interactive effects of multiple vernalization (*Vrn-1*)- and photoperiod (*Ppd-1*)-related genes on the growth habit of bread wheat and their association with heading and flowering time

**DOI:** 10.1186/s12870-018-1587-8

**Published:** 2018-12-27

**Authors:** Shulin Chen, Junsen Wang, Genwang Deng, Long Chen, Xiyong Cheng, Haixia Xu, Kehui Zhan

**Affiliations:** 1 College of Agronomy, Henan Agricultural University/Collaborative Innovation Center of Henan Grain Crops, Zhengzhou, 450002 China; 2 HuaGuan Seed Technology Co. Ltd., Zhoukou, Henan China; 3 YuLong Crops Research Institute, Xinzheng, Henan China

**Keywords:** Winter wheat, The yellow and Huai wheat production region (YHW), Growth habit, Growth period, Vernalization, Photoperiod

## Abstract

**Background:**

The precise identification of Winterness/Springness (growth habit) for bread wheat, which is determined by genes involved in vernalization and photoperiod, will contribute to the effective utilization of bread wheat varieties. Here, 198 varieties from the Yellow and Huai wheat production region (YHW) in China were collected to identify their vernalization (*Vrn-1*) and photoperiod (*Ppd-1*) gene composition via a series of functional markers and their association with vernalization and photoperiod requirements at three locations during two years of experiments. The growth habits were measured during the spring sowing season.

**Results:**

The results showed that the semi-winter varieties (grades1–4) were most prevalent in the population. The relative effects of single *Vrn* alleles on the growth period, such as heading date (HD) and/or flowering date (FD), were as follows: *Vrn-B1b* > *Vrn-B1a > Vrn-D1b* > *Vrn-D1a* > *vrn-D1* = *vrn-B1*. The interactive effects of *Vrn-B1* and *Vrn-D1* on HD and FD were identical to those of *Vrn-B1b*. Approximately 35.3% of the cultivars carried *Ppd-B1a* (photoperiod-insensitive) and exhibited the earliest HD and FD. The *Ppd-D1a-*insensitive allele (*Hapl II*) was carried by just 0.5% of the varieties; however, the other two sensitive alleles were present at a higher frequency, and their effects were slightly weaker than those of *Ppd-B1a*. In addition, strong interactive effects between *Ppd-B1* and *Ppd-D1* were detected. In terms of mean values among various genotypes, the effects followed the order of *Vrn-1* > *Ppd-1*.

**Conclusions:**

According to the results of ANOVA and least significant range (LSR) tests, we can conclude that *Vrn-1* rather than *Ppd-1* played a major role in controlling vernalization and photoperiod responses in this region. This research will be helpful for precisely characterizing and evaluating the HD, FD and even growth habit of varieties in the YHW at molecular levels.

**Electronic supplementary material:**

The online version of this article (10.1186/s12870-018-1587-8) contains supplementary material, which is available to authorized users.

## Background

Because of the ease of determining its optimum time for flowering and maturation, bread wheat (*Triticum aestivum* L., AABBDD, 2n = 42) is cultivated worldwide. Day length and low temperature act as environmental cues affecting the time to heading and flowering. The ability to perceive and respond to these signals is controlled by molecular pathways that regulate early growth habits in response to abiotic stress (*Vrn* alleles) and photoperiod (*Ppd* alleles) [1, 2].

In the past, the characterization of the growth habits of winter wheat in China, especially in the Yellow and Huai wheat production region, which is the largest winter wheat production region, relied on field evaluations or in-house artificial identification [3, 4]. However, these identification procedures are tedious and costly, and thus, the practicality of field evaluation and in-house artificial identification methods is limited. Furthermore, inconsistencies between breeders’ descriptions and government registrations sometimes occur due to inexact phenotypic identification methods. In contrast, molecular identification methods are relatively credible, but more novel types of vernalization alleles must be surveyed, and their interactive effects among each other remain unclear.

Indeed, the molecular basis for flowering time regulation has been extensively studied in wheat and other crops [5]. In hexaploid wheat, vernalization requirements are controlled by three major orthologous *Vrn*alleles—*Vrn-A1*, *Vrn-B1*, and *Vrn-D1*—which have been mapped onto the long arm of chromosomes 5A, 5B, and 5D, respectively [6–8]. Each of these loci encodes a MADS-box transcription factor orthologous to *AP1* in *Arabidopsis*, which is reported to be involved in floral meristem development during the transition from the vegetative phase to the reproductive phase [8]. The *VRN-1* gene is dominant for the spring growth habit, and it is upregulated by vernalization in winter lines [9, 10]. A homologue of the *Arabidopsis FT* gene, the *Vrn-3* gene, has been mapped to the short arm of chromosome 7 in wheat; this gene upregulated the *Vrn-1* genes and thus accelerated heading and flowering indirectly [11].

The emergence of dominant alleles at the *Vrn-A1* locus is a result of insertions and deletions within the promoter or a deletion within intron 1, which have been designated *Vrn-A1a*, *Vrn-A1b*, and *Vrn-A1c*, respectively [9, 10, 12, 13]. Spring growth habits can also be attributed to deletions at the *Vrn-B1* and *Vrn-D1* loci, which have been classified as insensitive vernalization types, and have been designated *Vrn-B1a* [10, 14, 15], *Vrn-D1a*, and *Vrn-D1b* [16]. The *Vrn-B1c* (novel) allele, which is due to the deletion of 0.8 kb and the duplication of 0.4 kb within intron 1, differs from *Vrn-B1a* [17, 18]. Another spring allele, *Vrn-B1b*, has also been described; this allele contains two deletions in the promoter region and is present in spring variety ‘Alpowa’ [14]. The various vernalization requirements of the *Vrn-1* alleles or combinations can result in variations in flowering time and spring growth habit [19]. In wheat and other temperate grasses, *VRN1* is also expressed in the leaves, where it acts as a repressor of *VRN2* [20, 21]. The detailed pathway of the vernalization genes involved in controlling wheat flowering was reviewed by Chen and Dubcovsky (2012) [20].

Photoperiod response is another vital factor affecting flowering time under long-day conditions. For wheat, photoperiod insensitivity (*Ppd-1a*) is widespread and especially prevalent in regions where crops grow during short days or when the crops mature before the onset of high summer temperatures [22]. Three semi-dominant orthologous *Ppd-1* loci—*Ppd-A1*, *Ppd-B1*, and *Ppd-D1*—have been mapped onto the short arm of chromosomes 2A, 2B, and 2D, respectively [23, 24]; these loci are all members of the *Pseudo-Response Regulator* (*PRR*) gene family, which is orthologous to the *Ppd-H1* gene family in barley [25]. A series of diagnostic markers have been used to efficiently screen for several variants [10, 13, 16, 24, 26].

The Yellow and Huai valley wheat production region (YHW) covers 45% of China’s total cultivation area but contributes 60–70% of the country’s wheat production. Varieties that flower and mature early are helpful in sustaining China’s double-harvest cropping system. In this study, we collected and identified a total of 198 popular varieties, elite lines, and landraces from China to (i) accurately identify the growth habits of the varieties via the field spring sowing method and evaluate their association with heading date (HD; growth period) and flowering date (FD; growth period) at three locations within the YHW during a two-year period (Zhengzhou, Zhumadian and Shangqiu in 2014 and 2015); (ii) use diagnostic molecular markers to determine the main allelic frequencies of *Vrn-1* and *Ppd-1*; and (iii) specifically determine the interactive effects between *Vrn-1* and *Ppd-1* allelic combinations on heading and flowering times. This study contributes knowledge concerning the effective selection of various types of growth habits of varieties and will be of service to the selection of early-maturation cultivars at the molecular level.

## Results

Semi-winter varieties were predominant in the YHW according to the field spring sowing method.

**Fig. 1:**
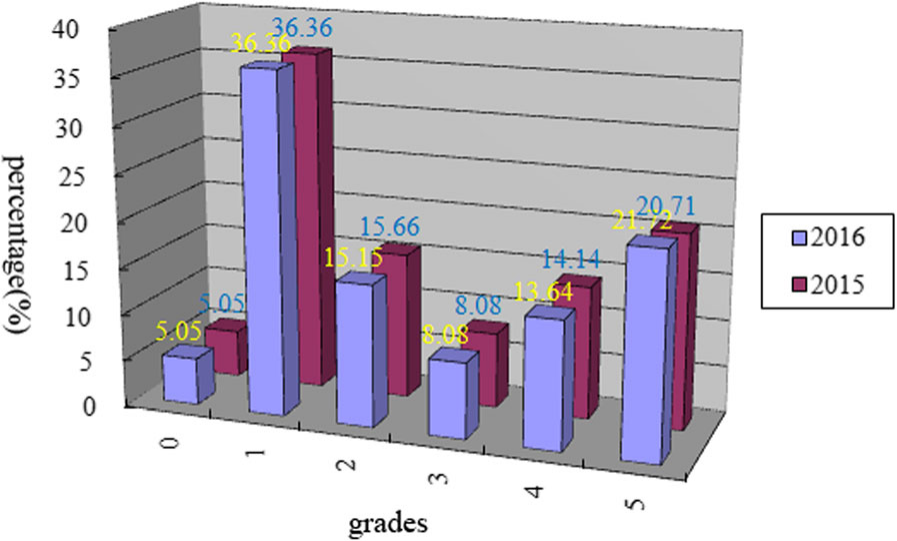
Distribution rate in each grade of winter/spring growth habits among 198 wheat materials in 2015 and 2016

The results of the two-year growth habits were very similar, and the order ranks recorded in 2015 strongly correlated with those recorded in 2016 (Pearson coefficient = 0.96). In general, the ranks of two accessions (Xinong979 and Yumai47) were inconsistent between years, but the discrepancies were only 1–2 grades. Our method separated 10 accessions (Yannong19, Beijing841, etc.) into winterness (grade 0) in 2015 and 2016, which accounted for 5.05%. One hundred and forty-seven accessions (74.24%) were identified as semi-winter types in 2015, and 145 (73.23%) were identified in 2016. In contrast, 41 (20.71%) and 43 (21.72%) grade 5 accessions belonged to the spring type in 2015 and 2016. Overall, the semi-winter varieties (grades1–4) were predominant in the YHW (Table 1; Fig. 1).

**Table 1:** The phenotypic variants in winter/spring growth habits and allelic variants on *Vrn-1* alleles

ID	Taxa	Registered	Origination	W/S_2016	W/S_2015	*Vrn-A1*	*Vrn-B1*	*Vrn-D1*	*Vrn-B3*
QZ01	Shanyou 225	winter	Shaanxi	5	5	*vrn-A1*	*vrn-B1*	*Vrn-D1a*	*vrn-B3*
QZ02	Aikang 58	semi-winter	Henan	3	3	*vrn-A1*	*vrn-B1*	*vrn-D1*	*vrn-B3*
QZ03	Zhoumai 24	semi-winter	Henan	2	2	*vrn-A1*	*vrn-B1*	*vrn-D1*	*vrn-B3*
QZ04	Zaoxiang 158	semi-winter	Henan	2	2	*vrn-A1*	*vrn-B1*	*vrn-D1*	*vrn-B3*
QZ05	Zhengmai 366	semi-winter	Henan	2	2	*vrn-A1*	*vrn-B1*	*vrn-D1*	*vrn-B3*
QZ06	Bainong 418	semi-winter	Henan	4	4	*vrn-A1*	*vrn-B1*	*vrn-D1*	*vrn-B3*
QZ07	Taihemai 1	semi-winter	Henan	1	1	*vrn-A1*	*vrn-B1*	*vrn-D1*	*vrn-B3*
QZ08	Anmai 8	semi-winter	Henan	1	1	*vrn-A1*	*vrn-B1*	*vrn-D1*	*vrn-B3*
QZ09	Huaimai 05159	semi-winter	Henan	4	4	*vrn-A1*	*vrn-B1*	*vrn-D1*	*vrn-B3*
QZ10	Zhoumai 16	semi-winter	Henan	1	1	*vrn-A1*	*vrn-B1*	*vrn-D1*	*vrn-B3*
QZ11	Xinmai 18	semi-winter	Henan	1	1	*vrn-A1*	*vrn-B1*	*vrn-D1*	*vrn-B3*
QZ12	Xianmai 13	weak spring	Henan	5	5	*vrn-A1*	*vrn-B1*	*Vrn-D1a*	*vrn-B3*
QZ13	Yandian 9433	semi-winter	Henan	1	1	*vrn-A1*	*vrn-B1*	*vrn-D1*	*vrn-B3*
QZ14	Zhongchuang 805	weak spring	Henan	1	1	*vrn-A1*	*vrn-B1*	*vrn-D1*	*vrn-B3*
QZ15	Zhengyumai 9987	semi-winter	Henan	3	3	*vrn-A1*	*vrn-B1*	*Vrn-D1a*	*vrn-B3*
QZ16	Luomai 31	semi-winter	Henan	3	3	*vrn-A1*	*vrn-B1*	*vrn-D1*	*vrn-B3*
QZ17	Yimai 6	semi-winter	Henan	2	2	*vrn-A1*	*vrn-B1*	*Vrn-D1b*	*vrn-B3*
QZ18	Zhengzhong 17	semi-winter	Henan	3	3	*vrn-A1*	*vrn-B1*	*Vrn-D1a*	*vrn-B3*
QZ19	Fanmai 803	semi-winter	Henan	1	1	*vrn-A1*	*vrn-B1*	*vrn-D1*	*vrn-B3*
QZ20	Zhoumai 27	semi-winter	Henan	2	2	*vrn-A1*	*vrn-B1*	*vrn-D1*	*vrn-B3*
QZ21	Luomai 28	semi-winter	Henan	4	4	*vrn-A1*	*vrn-B1*	*vrn-D1*	*vrn-B3*
QZ22	Yunong 982	semi-winter	Henan	1	1	*vrn-A1*	*vrn-B1*	*vrn-D1*	*vrn-B3*
QZ23	Zhengmai 1023	semi-winter	Henan	0	0	*vrn-A1*	*vrn-B1*	*vrn-D1*	*vrn-B3*
QZ24	BN 160	semi-winter	Henan	1	1	*vrn-A1*	*vrn-B1*	*vrn-D1*	*vrn-B3*
QZ25	04 zhong 36	weak spring	Henan	5	5	*vrn-A1*	*vrn-B1*	*Vrn-D1a*	*vrn-B3*
QZ26	Lankao198	weak spring	Henan	5	5	*vrn-A1*	*vrn-B1*	*Vrn-D1b*	*vrn-B3*
QZ27	Fengdecunmai 5	semi-winter	Henan	3	3	*vrn-A1*	*vrn-B1*	*vrn-D1*	*vrn-B3*
QZ28	Guoyu 101	semi-winter	Henan	1	1	*vrn-A1*	*vrn-B1*	*vrn-D1*	*vrn-B3*
QZ29	Wen 0418	semi-winter	Henan	1	1	*vrn-A1*	*vrn-B1*	*vrn-D1*	*vrn-B3*
QZ30	Yunong 202	semi-winter	Henan	4	4	*vrn-A1*	*vrn-B1*	*vrn-D1*	*vrn-B3*
QZ31	Zhongyu 9307	semi-winter	Henan	1	1	*vrn-A1*	*vrn-B1*	*vrn-D1*	*vrn-B3*
QZ32	Ruzhou 0319	semi-winter	Henan	2	2	*vrn-A1*	*vrn-B1*	*vrn-D1*	*vrn-B3*
QZ33	Yumai 41	semi-winter	Henan	0	0	*vrn-A1*	*vrn-B1*	*vrn-D1*	*vrn-B3*
QZ34	Yumai 55	semi-winter	Henan	1	1	*vrn-A1*	*vrn-B1*	*vrn-D1*	*vrn-B3*
QZ35	Yunong 186	semi-winter	Henan	4	4	*vrn-A1*	*vrn-B1*	*vrn-D1*	*vrn-B3*
QZ36	Junda 106	semi-winter	Henan	1	1	*vrn-A1*	*vrn-B1*	*vrn-D1*	*vrn-B3*
QZ37	Yumai 49	semi-winter	Henan	1	1	*vrn-A1*	*vrn-B1*	*vrn-D1*	*vrn-B3*
QZ38	Zhengmai 7698	semi-winter	Henan	4	4	*vrn-A1*	*vrn-B1*	*vrn-D1*	*vrn-B3*
QZ39	Lunxuan 1298	semi-winter	Henan	1	1	*vrn-A1*	*vrn-B1*	*vrn-D1*	*vrn-B3*
QZ40	Mengmai 023	weak spring	Henan	5	5	*vrn-A1*	*Vrn-B1a*	*vrn-D1*	*vrn-B3*
QZ41	Luomai 23	semi-winter	Henan	5	5	*vrn-A1*	*vrn-B1*	*Vrn-D1a*	*vrn-B3*
QZ42	Jiyanmai 7	semi-winter	Henan	2	2	*vrn-A1*	*vrn-B1*	*vrn-D1*	*vrn-B3*
QZ43	Fengdecunmai 8	semi-winter	Henan	1	1	*vrn-A1*	*vrn-B1*	*vrn-D1*	*vrn-B3*
QZ44	Zhoumai 9	semi-winter	Henan	1	1	*vrn-A1*	*vrn-B1*	*vrn-D1*	*vrn-B3*
QZ45	Zhengyumai 0519	semi-winter	Henan	1	1	*vrn-A1*	*vrn-B1*	*vrn-D1*	*vrn-B3*
QZ46	Xuke 316	semi-winter	Henan	4	4	*vrn-A1*	*vrn-B1*	*Vrn-D1a*	*vrn-B3*
QZ47	Luomai 18	weak spring	Henan	5	5	*vrn-A1*	*vrn-B1*	*Vrn-D1a*	*vrn-B3*
QZ48	Xinong 979	semi-winter	Henan	5	4	*vrn-A1*	*vrn-B1*	*vrn-D1*	*vrn-B3*
QZ49	Cunmai 11	semi-winter	Henan	2	2	*vrn-A1*	*vrn-B1*	*vrn-D1*	*vrn-B3*
QZ50	Fengdecunmai 12	semi-winter	Henan	2	2	*vrn-A1*	*vrn-B1*	*vrn-D1*	*vrn-B3*
QZ51	Huaichuan 919	semi-winter	Henan	2	2	*vrn-A1*	*vrn-B1*	*Vrn-D1a*	*vrn-B3*
QZ52	Zou 8425B	semi-winter	Henan	4	4	*vrn-A1*	*vrn-B1*	*Vrn-D1a*	*vrn-B3*
QZ53	Yunong 211	semi-winter	Henan	1	1	*vrn-A1*	*vrn-B1*	*vrn-D1*	*vrn-B3*
QZ54	LS 6109	semi-winter	Shandong	1	1	*vrn-A1*	*vrn-B1*	*vrn-D1*	*vrn-B3*
QZ55	Lankao 182	semi-winter	Henan	1	1	*vrn-A1*	*vrn-B1*	*vrn-D1*	*vrn-B3*
QZ56	Xumai 1242	semi-winter	Henan	2	2	*vrn-A1*	*vrn-B1*	*vrn-D1*	*vrn-B3*
QZ57	Zhoumai 26	semi-winter	Henan	5	5	*vrn-A1*	*vrn-B1*	*vrn-D1*	*vrn-B3*
QZ58	Yamai 1	weak spring	Henan	5	5	*vrn-A1*	*Vrn-B1a*	*vrn-D1*	*vrn-B3*
QZ59	Yujiao 5	semi-winter	Henan	0	0	*vrn-A1*	*vrn-B1*	*vrn-D1*	*vrn-B3*
QZ60	Fanmai 11	semi-winter	Henan	4	4	*vrn-A1*	*vrn-B1*	*vrn-D1*	*vrn-B3*
QZ61	Zhengmai 103	semi-winter	Henan	4	4	*vrn-A1*	*vrn-B1*	*Vrn-D1a*	*vrn-B3*
QZ62	Pumai 053	semi-winter	Henan	1	1	*vrn-A1*	*vrn-B1*	*vrn-D1*	*vrn-B3*
QZ63	Tunfeng 802	semi-winter	Henan	3	3	*vrn-A1*	*vrn-B1*	*vrn-D1*	*vrn-B3*
QZ64	Fengdecunmai 1	semi-winter	Henan	4	4	*vrn-A1*	*vrn-B1*	*vrn-D1*	*vrn-B3*
QZ65	Pu2056	semi-winter	Henan	5	5	*vrn-A1*	*Vrn-B1b*	*vrn-D1*	*vrn-B3*
QZ66	Xinmai 19	semi-winter	Henan	4	4	*vrn-A1*	*vrn-B1*	*vrn-D1*	*vrn-B3*
QZ67	Wenliang 1	semi-winter	Henan	1	1	*vrn-A1*	*vrn-B1*	*vrn-D1*	*vrn-B3*
QZ68	Zhongyu 9302	semi-winter	Henan	1	1	*vrn-A1*	*vrn-B1*	*vrn-D1*	*vrn-B3*
QZ69	Zhengmai 583	semi-winter	Henan	1	1	*vrn-A1*	*vrn-B1*	*vrn-D1*	*vrn-B3*
QZ70	Hengguan 35	semi-winter	Henan	5	5	*vrn-A1*	*vrn-B1*	*Vrn-D1b*	*vrn-B3*
QZ71	Luomai 24	weak spring	Henan	5	5	*vrn-A1*	*vrn-B1*	*Vrn-D1a*	*vrn-B3*
QZ72	Yunong 416	semi-winter	Henan	5	5	*vrn-A1*	*vrn-B1*	*vrn-D1*	*vrn-B3*
QZ73	Zhoumai 22	semi-winter	Henan	1	1	*vrn-A1*	*vrn-B1*	*vrn-D1*	*vrn-B3*
QZ74	Yumai 52	semi-winter	Henan	1	1	*vrn-A1*	*vrn-B1*	*vrn-D1*	*vrn-B3*
QZ75	FS 059	semi-winter	Henan	1	1	*vrn-A1*	*vrn-B1*	*vrn-D1*	*vrn-B3*
QZ76	Pingan 11	semi-winter	Henan	1	1	*vrn-A1*	*vrn-B1*	*vrn-D1*	*vrn-B3*
QZ77	Bonong 6	weak spring	Henan	5	5	*vrn-A1*	*vrn-B1*	*vrn-D1*	*vrn-B3*
QZ78	Zhoumai 18	semi-winter	Henan	1	1	*vrn-A1*	*vrn-B1*	*vrn-D1*	*vrn-B3*
QZ79	Xuke 168	semi-winter	Henan	3	3	*vrn-A1*	*vrn-B1*	*Vrn-D1a*	*vrn-B3*
QZ80	Pumai 10	semi-winter	Henan	3	3	*vrn-A1*	*vrn-B1*	*Vrn-D1a*	*vrn-B3*
QZ81	Xumai 0054	semi-winter	Jiangsu	1	1	*vrn-A1*	*vrn-B1*	*vrn-D1*	*vrn-B3*
QZ82	Zhengyou 6	semi-winter	Henan	5	5	*vrn-A1*	*Vrn-B1a*	*vrn-D1*	*vrn-B3*
QZ83	Yunong 9901	weak spring	Henan	5	5	*vrn-A1*	*vrn-B1*	*Vrn-D1a*	*vrn-B3*
QZ84	Zhengmai 0856	semi-winter	Henan	4	4	*vrn-A1*	*vrn-B1*	*vrn-D1*	*vrn-B3*
QZ85	Luomai 8	semi-winter	Henan	1	1	*vrn-A1*	*vrn-B1*	*vrn-D1*	*vrn-B3*
QZ86	Zhoumai 13	semi-winter	Henan	2	2	*vrn-A1*	*vrn-B1*	*Vrn-D1a*	*vrn-B3*
QZ87	Luo 10 T07	semi-winter	Henan	2	2	*vrn-A1*	*vrn-B1*	*vrn-D1*	*vrn-B3*
QZ88	Yuanyu 3	weak spring	Henan	5	5	*vrn-A1*	*vrn-B1*	*Vrn-D1a*	*vrn-B3*
QZ89	Fengdecunmai 10	semi-winter	Henan	4	4	*vrn-A1*	*vrn-B1*	*vrn-D1*	*vrn-B3*
QZ90	Hongmai 118	semi-winter	Henan	1	1	*vrn-A1*	*vrn-B1*	*vrn-D1*	*vrn-B3*
QZ91	Yumai 14 You	semi-winter	Henan	4	4	*vrn-A1*	*vrn-B1*	*Vrn-D1a*	*vrn-B3*
QZ92	Zhoumai 19	semi-winter	Henan	4	4	*vrn-A1*	*vrn-B1*	*Vrn-D1b*	*vrn-B3*
QZ93	Guomai 301	semi-winter	Henan	0	0	*vrn-A1*	*vrn-B1*	*vrn-D1*	*vrn-B3*
QZ94	Zhengmai 113	weak spring	Henan	5	5	*vrn-A1*	*vrn-B1*	*Vrn-D1a*	*vrn-B3*
QZ95	Pumai 9	weak spring	Henan	5	5	*vrn-A1*	*vrn-B1*	*Vrn-D1a*	*vrn-B3*
QZ96	Pingan 8	semi-winter	Henan	1	1	*vrn-A1*	*vrn-B1*	*vrn-D1*	*vrn-B3*
QZ97	Xinmai 20	weak spring	Henan	5	5	*vrn-A1*	*vrn-B1*	*Vrn-D1a*	*vrn-B3*
QZ98	Zhongmai 875	semi-winter	Beijing	2	2	*vrn-A1*	*vrn-B1*	*vrn-D1*	*vrn-B3*
QZ99	Zhongyanmai 0708	weak spring	Jiangsu	3	3	*vrn-A1*	*vrn-B1*	*vrn-D1*	*vrn-B3*
QZ100	Zhongmai 1	semi-winter	Henan	1	1	*vrn-A1*	*vrn-B1*	*vrn-D1*	*vrn-B3*
QZ101	Nongda 1108	semi-winter	Beijing	1	1	*vrn-A1*	*vrn-B1*	*Vrn-D1a*	*vrn-B3*
QZ102	Yumai 47	weak spring	Henan	5	2	*vrn-A1*	*vrn-B1*	*vrn-D1*	*vrn-B3*
QZ103	Zhengmai 98	semi-winter	Henan	2	2	*vrn-A1*	*vrn-B1*	*Vrn-D1a*	*vrn-B3*
QZ104	Xinong 889	semi-winter	Shaanxi	2	2	*vrn-A1*	*vrn-B1*	*vrn-D1*	*vrn-B3*
QZ105	Neixiang 188	semi-winter	Henan	2	2	*vrn-A1*	*vrn-B1*	*vrn-D1*	*vrn-B3*
QZ106	Jimai 22	semi-winter	Shandong	3	3	*vrn-A1*	*vrn-B1*	*vrn-D1*	*vrn-B3*
QZ107	Fanmai 5	semi-winter	Henan	2	2	*vrn-A1*	*vrn-B1*	*Vrn-D1a*	*vrn-B3*
QZ108	Huayu 198	semi-winter	Henan	0	0	*vrn-A1*	*vrn-B1*	*vrn-D1*	*vrn-B3*
QZ109	Huangming 116	weak spring	Henan	3	3	*vrn-A1*	*vrn-B1*	*vrn-D1*	*vrn-B3*
QZ110	Zhengmai 9694	semi-winter	Henan	1	1	*vrn-A1*	*vrn-B1*	*vrn-D1*	*vrn-B3*
QZ111	Yunong 949	weak spring	Henan	5	5	*vrn-A1*	*vrn-B1*	*Vrn-D1a*	*vrn-B3*
QZ112	Shan 160	semi-winter	Shaanxi	3	3	*vrn-A1*	*vrn-B1*	*vrn-D1*	*vrn-B3*
QZ113	Luomai 4	semi-winter	Henan	1	1	*vrn-A1*	*vrn-B1*	*vrn-D1*	*vrn-B3*
QZ114	Luoxin 998	semi-winter	Henan	1	1	*vrn-A1*	*vrn-B1*	*Vrn-D1a*	*vrn-B3*
QZ115	Yunong 201	semi-winter	Henan	1	1	*vrn-A1*	*vrn-B1*	*vrn-D1*	*vrn-B3*
QZ116	Kaimai 21	semi-winter	Henan	1	1	*vrn-A1*	*vrn-B1*	*vrn-D1*	*vrn-B3*
QZ117	Yubao 1	semi-winter	Henan	1	1	*vrn-A1*	*vrn-B1*	*Vrn-D1b*	*vrn-B3*
QZ118	Bainong 207	semi-winter	Henan	2	2	*vrn-A1*	*vrn-B1*	*vrn-D1*	*vrn-B3*
QZ119	Luomai 21	semi-winter	Henan	3	3	*vrn-A1*	*vrn-B1*	*Vrn-D1b*	*vrn-B3*
QZ120	Zhengmai 101	weak spring	Henan	1	1	*vrn-A1*	*vrn-B1*	*vrn-D1*	*vrn-B3*
QZ121	Xinmai 9	semi-winter	Henan	1	1	*vrn-A1*	*vrn-B1*	*vrn-D1*	*vrn-B3*
QZ122	Xinmai 208	weak spring	Henan	5	5	*vrn-A1*	*vrn-B1*	*Vrn-D1b*	*vrn-B3*
QZ123	Yumai 34	weak spring	Henan	5	5	*vrn-A1*	*Vrn-B1b*	*vrn-D1*	*vrn-B3*
QZ124	Kaimai 18	semi-winter	Henan	2	2	*vrn-A1*	*vrn-B1*	*vrn-D1*	*vrn-B3*
QZ125	Xuke 793	semi-winter	Henan	1	1	*vrn-A1*	*vrn-B1*	*vrn-D1*	*vrn-B3*
QZ126	Fanmai 7030	semi-winter	Henan	4	4	*vrn-A1*	*vrn-B1*	*vrn-D1*	*vrn-B3*
QZ127	Shi 4185	semi-winter	Hebei	3	3	*vrn-A1*	*vrn-B1*	*Vrn-D1a*	*vrn-B3*
QZ128	Xin 0208	semi-winter	Henan	2	2	*vrn-A1*	*vrn-B1*	*vrn-D1*	*vrn-B3*
QZ129	Zhengmai 379	semi-winter	Henan	3	3	*vrn-A1*	*vrn-B1*	*Vrn-D1a*	*vrn-B3*
QZ130	Jimai 20	semi-winter	Shandong	4	4	*vrn-A1*	*vrn-B1*	*vrn-D1*	*vrn-B3*
QZ131	Qiule 2122	semi-winter	Henan	4	4	*vrn-A1*	*vrn-B1*	*Vrn-D1a*	*vrn-B3*
QZ132	Zhoumai 23	weak spring	Henan	4	4	*vrn-A1*	*vrn-B1*	*Vrn-D1a*	*vrn-B3*
QZ133	Zhengyumai 043	semi-winter	Henan	1	1	*vrn-A1*	*vrn-B1*	*Vrn-D1a*	*vrn-B3*
QZ134	Zhengmai 004	semi-winter	Henan	3	3	*vrn-A1*	*vrn-B1*	*Vrn-D1a*	*vrn-B3*
QZ135	Wennong 14	semi-winter	Shandong	2	2	*vrn-A1*	*vrn-B1*	*vrn-D1*	*vrn-B3*
QZ136	Xinhan 1	semi-winter	Henan	4	4	*vrn-A1*	*vrn-B1*	*Vrn-D1a*	*vrn-B3*
QZ137	Yumai 18	weak spring	Henan	5	5	*vrn-A1*	*vrn-B1*	*Vrn-D1a*	*vrn-B3*
QZ138	Xuke 415	semi-winter	Henan	1	1	*vrn-A1*	*vrn-B1*	*vrn-D1*	*vrn-B3*
QZ139	Pingan 9	semi-winter	Henan	1	1	*vrn-A1*	*vrn-B1*	*vrn-D1*	*vrn-B3*
QZ140	Shangmai 156	semi-winter	Henan	1	1	*vrn-A1*	*vrn-B1*	*Vrn-D1a*	*vrn-B3*
QZ141	Xinong 9871	semi-winter	Shaanxi	5	5	*vrn-A1*	*vrn-B1*	*Vrn-D1a*	*vrn-B3*
QZ142	Pingan 3	semi-winter	Henan	1	1	*vrn-A1*	*vrn-B1*	*vrn-D1*	*vrn-B3*
QZ143	Zhongyu 12	semi-winter	Henan	2	2	*vrn-A1*	*vrn-B1*	*Vrn-D1b*	*vrn-B3*
QZ144	Yumai 58	semi-winter	Henan	1	1	*vrn-A1*	*vrn-B1*	*vrn-D1*	*vrn-B3*
QZ145	Xun 9917	semi-winter	Henan	4	4	*vrn-A1*	*vrn-B1*	*Vrn-D1b*	*vrn-B3*
QZ146	Ping’an 6	weak spring	Henan	5	5	*vrn-A1*	*Vrn-B1b*	*Vrn-D1a*	*vrn-B3*
QZ147	Xinmai 26	semi-winter	Henan	1	1	*vrn-A1*	*vrn-B1*	*vrn-D1*	*vrn-B3*
QZ148	Lankaoaizao 8	weak spring	Henan	2	2	*vrn-A1*	*vrn-B1*	*vrn-D1*	*vrn-B3*
QZ149	Fengyou 6	weak spring	Henan	5	5	*vrn-A1*	*Vrn-B1a*	*vrn-D1*	*vrn-B3*
QZ150	Huaimai 0882	semi-winter	Jiangsu	1	1	*vrn-A1*	*vrn-B1*	*vrn-D1*	*vrn-B3*
QZ151	Taikong 6	weak spring	Henan	1	1	*vrn-A1*	*vrn-B1*	*vrn-D1*	*vrn-B3*
QZ152	Xinong 529	weak spring	Shaanxi	5	5	*vrn-A1*	*vrn-B1*	*Vrn-D1a*	*vrn-B3*
QZ153	Yumai 51	weak spring	Henan	5	5	*vrn-A1*	*vrn-B1*	*Vrn-D1a*	*vrn-B3*
QZ154	Luo 6073	semi-winter	Henan	1	1	*vrn-A1*	*vrn-B1*	*vrn-D1*	*vrn-B3*
QZ155	Bainong 64	semi-winter	Henan	1	1	*vrn-A1*	*vrn-B1*	*vrn-D1*	*vrn-B3*
QZ156	Jinan 17	winter	Shandong	2	2	*vrn-A1*	*vrn-B1*	*vrn-D1*	*vrn-B3*
QZ157	Yanzhan 4110	weak spring	Henan	5	5	*vrn-A1*	*vrn-B1*	*Vrn-D1a*	*vrn-B3*
QZ158	Weilai 0818	semi-winter	Anhui	1	1	*vrn-A1*	*vrn-B1*	*vrn-D1*	*vrn-B3*
QZ159	Yanke 028	weak spring	Henan	5	5	*vrn-A1*	*Vrn-B1a*	*vrn-D1*	*vrn-B3*
QZ160	86(79)-128	semi-winter	Henan	4	4	*vrn-A1*	*vrn-B1*	*vrn-D1*	*vrn-B3*
QZ161	Yanmai864	weak spring	Henan	5	5	*vrn-A1*	*Vrn-B1b*	*vrn-D1*	*vrn-B3*
QZ162	Xiaoyan 81	semi-winter	Beijing	2	2	*vrn-A1*	*vrn-B1*	*vrn-D1*	*vrn-B3*
QZ163	Liangxing 99	semi-winter	Shandong	2	2	*vrn-A1*	*vrn-B1*	*vrn-D1*	*vrn-B3*
QZ164	Tainong 8968	semi-winter	Shandong	0	0	*vrn-A1*	*vrn-B1*	*vrn-D1*	*vrn-B3*
QZ165	Huayumai 118	semi-winter	Henan	2	2	*vrn-A1*	*vrn-B1*	*vrn-D1*	*vrn-B3*
QZ166	Yanshi 16	weak spring	Henan	5	5	*vrn-A1*	*vrn-B1*	*Vrn-D1a*	*vrn-B3*
QZ167	Xuke 1	semi-winter	Henan	1	1	*vrn-A1*	*vrn-B1*	*Vrn-D1a*	*vrn-B3*
QZ168	Zhoumai 30	semi-winter	Henan	1	1	*vrn-A1*	*vrn-B1*	*Vrn-D1b*	*vrn-B3*
QZ169	Yunong 4023	semi-winter	Henan	1	1	*vrn-A1*	*vrn-B1*	*vrn-D1*	*vrn-B3*
QZ170	Zhengpinmai 8	semi-winter	Henan	1	1	*vrn-A1*	*vrn-B1*	*vrn-D1*	*vrn-B3*
QZ171	Zhengmai 9023	weak spring	Henan	5	5	*vrn-A1*	*Vrn-B1b*	*vrn-D1*	*vrn-B3*
QZ172	Shiluan 02–1	semi-winter	Hebei	0	0	*vrn-A1*	*vrn-B1*	*vrn-D1*	*vrn-B3*
QZ173	Aifeng 3	winter	Shaanxi	1	1	*vrn-A1*	*vrn-B1*	*Vrn-D1b*	*vrn-B3*
QZ174	Han 6172	winter	Hebei	2	2	*vrn-A1*	*vrn-B1*	*vrn-D1*	*vrn-B3*
QZ175	Luohan 3	semi-winter	Henan	4	4	*vrn-A1*	*vrn-B1*	*vrn-D1*	*vrn-B3*
QZ176	Yannong 19	winter	Shandong	0	0	*vrn-A1*	*vrn-B1*	*vrn-D1*	*vrn-B3*
QZ177	Huaimai 19	weak spring	Jiangsu	1	1	*vrn-A1*	*vrn-B1*	*vrn-D1*	*vrn-B3*
QZ178	Yanmai 8911	semi-winter	Shaanxi	1	1	*vrn-A1*	*vrn-B1*	*Vrn-D1b*	*vrn-B3*
QZ179	Bainong 3217	weak winter	Henan	5	5	*vrn-A1*	*vrn-B1*	*Vrn-D1a*	*vrn-B3*
QZ180	Yumai 68	semi-winter	Henan	1	1	*vrn-A1*	*vrn-B1*	*vrn-D1*	*vrn-B3*
QZ181	Yumai 2	semi-winter	Henan	1	1	*vrn-A1*	*vrn-B1*	*vrn-D1*	*vrn-B3*
QZ182	Gaocheng 8901	semi-winter	Hebei	1	1	*vrn-A1*	*vrn-B1*	*Vrn-D1a*	*vrn-B3*
QZ183	Huarui 00712	semi-winter	Jiangsu	5	5	*vrn-A1*	*vrn-B1*	*Vrn-D1a*	*vrn-B3*
QZ184	Shannong 7859	semi-winter	Shaanxi	4	4	*vrn-A1*	*vrn-B1*	*vrn-D1*	*vrn-B3*
QZ185	Xiaoyan 22	weak spring	Shaanxi	1	1	*vrn-A1*	*vrn-B1*	*Vrn-D1b*	*vrn-B3*
QZ186	Ruiquanmai 168	semi-winter	Henan	4	4	*vrn-A1*	*vrn-B1*	*Vrn-D1b*	*vrn-B3*
QZ187	Hemai 26	semi-winter	Henan	0	0	*vrn-A1*	*vrn-B1*	*vrn-D1*	*vrn-B3*
QZ188	Yan 99,102	semi-winter	Shandong	2	2	*vrn-A1*	*vrn-B1*	*Vrn-D1b*	*vrn-B3*
QZ189	Jihan 2	semi-winter	Shandong	5	5	*vrn-A1*	*vrn-B1*	*Vrn-D1b*	*vrn-B3*
QZ190	Yumai 4	weak spring	Henan	4	4	*vrn-A1*	*vrn-B1*	*vrn-D1*	*vrn-B3*
QZ191	Luohan 6	semi-winter	Henan	5	5	*vrn-A1*	*vrn-B1*	*vrn-D1*	*vrn-B3*
QZ192	Zhenghan 1	semi-winter	Henan	1	1	*vrn-A1*	*vrn-B1*	*vrn-D1*	*vrn-B3*
QZ193	Jinmai 47	semi-winter	Shanxi	1	1	*vrn-A1*	*vrn-B1*	*vrn-D1*	*vrn-B3*
QZ194	Yumai 8	semi-winter	Henan	1	1	*vrn-A1*	*vrn-B1*	*vrn-D1*	*vrn-B3*
QZ195	Beijing 841	winter	Beijing	0	0	*vrn-A1*	*vrn-B1*	*vrn-D1*	*vrn-B3*
QZ196	Sumai 3	weak spring	Jiangsu	5	5	*vrn-A1*	*vrn-B1*	*Vrn-D1a*	*vrn-B3*
QZ197	Nanda 2419	weak spring	Jiangsu	5	5	*vrn-A1*	*vrn-B1*	*Vrn-D1a*	*vrn-B3*
QZ198	Chinese Spring	spring	Sichuan	5	5	*vrn-A1*	*vrn-B1*	*Vrn-D1a*	*vrn-B3*

W/S means winter/spring growth habits in 2015 or 2016

In comparison with the data on regional trials (registered, Table 1), our data showed that the consistency was 89.39% when the accessions were divided into winter and spring groups, although 21 varieties need to be re-examined. In detail, of the varieties identified in the winter group, 10 varieties, including Huangming116, Lankaoaizao8, Yumai4, Taikong6, Zhengmai101, Zhongchuang805, Zhoumai23, Zhongyanmai 0708, Huaimai 19 and Xiaoyan 22, qualified as week spring (spring) type according to the registered results. The 11 spring growth habit accessions, which included Jihan2, Hengguan35, Zhengyou6, Luohan6, Luomai23, Pu2056, Shanyou225, Xinong9871, Yunong416, Zhoumai26 and Huarui 00712, were misclassified as accessions having winter (or semi-winter) growth habits. The reason is that these varieties were registered ten years ago, and winter-spring identification was not evaluated during registration tests at that time.

### Growth habits were highly associated with growth periods in six environments

**Fig. 2:**
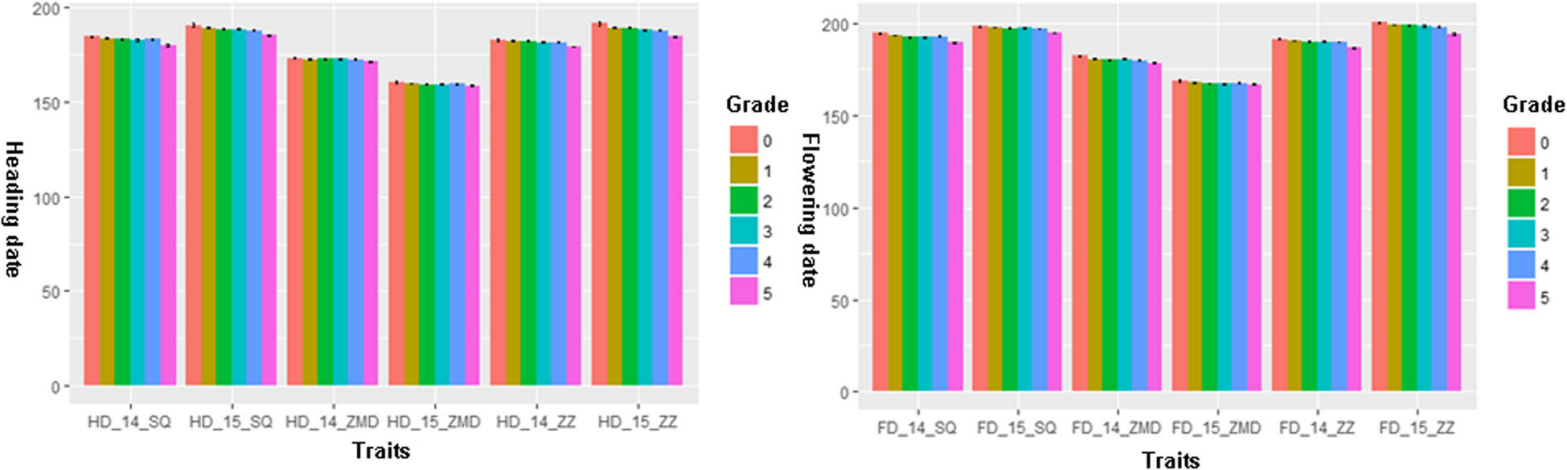
Correlations between growth habits and heading and flowering date (HD and FD) in six environments

The results of the joint ANOVA analysis revealed that significant differences in the mean values of the HD or FD grouped by grades 0–5 in all six environments (Table 2). Briefly, grade 5 exhibited the shortest length of the growth period, while grade 0 exhibited the greatest length. In detail, significant differences in the average values of growth period data were also found between different levels of each trait. The trend was similar to that revealed by the joint ANOVA results. Generally, the smaller the value of the HD or FD is, the greater the value of the grade (Fig. 2).

**Table 2:** The joint ANOVA analysis in HD and FD grouped by growth habit in six environments

Traits	0	1	2	3	4	5	r
HD_14_SQ	183.3 ± 2.0(a)	182.3 ± 2.2(ab)	181.9 ± 2.2(ab)	181.3 ± 1.9(b)	181.7 ± 2.9(ab)	178.5 ± 2.9(c)	−0.871*
HD_15_SQ	189.3 ± 2.4(a)	187.9 ± 2.0(b)	186.9 ± 2.0(b)	187.1 ± 2.0(b)	186.5 ± 2.1(b)	183.7 ± 2.2(c)	−0.922**
HD_14_ZMD	172.0 ± 1.8(a)	171.3 ± 1.4(a)	171.3 ± 1.2(a)	171.3 ± 1.7(a)	171.0 ± 1.7(a)	169.9 ± 1.1(b)	−0.884**
HD_15_ZMD	159.1 ± 1.5(a)	158.3 ± 1.1(b)	157.9 ± 1.5(bc)	157.9 ± 1.1(bc)	158.1 ± 1.2(b)	157.2 ± 1.5(c)	−0.869*
HD_14_ZZ	181.7 ± 1.9(a)	180.9 ± 1.1(ab)	180.8 ± 1.3(ab)	180.1 ± 1.3(b)	180.1 ± 1.5(b)	177.6 ± 1.6(c)	−0.897**
HD_15_ZZ	190.2 ± 3.5(a)	187.9 ± 2.3(b)	187.6 ± 1.8(bc)	186.8 ± 1.8(bc)	186.4 ± 1.9(c)	183.1 ± 2.2(d)	−0.938**
HD_average	180.3 ± 11.4(a)	179.1 ± 10.7(a)	178.8 ± 10.7(a)	178.4 ± 10.4(ab)	178.3 ± 10.3(ab)	176.0 ± 9.5(b)	−0.915**
FD_14_SQ	193.7 ± 1.8(a)	192.2 ± 1.8(b)	191.4 ± 2.0(b)	191.5 ± 1.5(b)	191.9 ± 2.6(b)	188.3 ± 2.5(c)	−0.838*
FD_15_SQ	198.4 ± 1.0(a)	197.5 ± 1.0(b)	197.1 ± 0.8(b)	197.4 ± 0.7(b)	196.9 ± 1.0(b)	194.8 ± 1.4(c)	−0.867*
FD_14_ZMD	181.3 ± 1.0(a)	179.5 ± 1.8(b)	179.2 ± 1.8(b)	179.5 ± 1.8(b)	178.7 ± 1.9(b)	177.0 ± 1.8(c)	−0.907**
FD_15_ZMD	167.4 ± 2.1(a)	166.5 ± 1.5(ab)	166.1 ± 1.4(b)	165.9 ± 1.4(b)	166.4 ± 1.6(ab)	165.6 ± 1.6(b)	−0.813*
FD_14_ZZ	190.6 ± 1.6(a)	189.5 ± 1.4(ab)	189.0 ± 1.5(b)	188.8 ± 1.3(b)	188.6 ± 1.3(b)	185.4 ± 1.9(c)	−0.885**
FD_15_ZZ	199.3 ± 1.7(a)	198.2 ± 1.6(ab)	197.9 ± 1.6(bc)	197.3 ± 1.6(bc)	196.8 ± 1.6(c)	192.9 ± 2.6(d)	−0.889**
FD_average	189.1 ± 11.4(a)	188.1 ± 11.2(a)	187.6 ± 11.3(ab)	187.6 ± 11.1(ab)	187.4 ± 11.0(ab)	184.9 ± 10.2(b)	−0.886**

Lowercase letters indicate significant differences at the 0.05 level; * and ** indicates significant differences at the 0.05 and 0.01 level, respectively. The correlation coefficients (r) were calculated between the mean values of each grade and corresponding rank (r_0.05,5_ = 0.754 and r_0.01,5_ = 0.875); the ranks of the growth habit were calculated via the arithmetic means obtained during a two-year period, and all the data of all discrepant individuals were omitted

Significant negative correlations were detected between growth habit and growth period in six environments (Table 2, *p* < 0.01). According to the results of joint variance analysis, the mean values of HD and FD were also correlated with growth habit; the Pearson correlation coefficients were − 0.915 and − 0.886, respectively. Generally, these results also indicated that HD were more tightly, though negatively, related with growth habits. Furthermore, the range of correlation coefficients in six environments were from −0.813 (FD_15_ZMD) to −0.938 (HD_15_ZZ). Thus, we could conclude that the duration of the heading and flowering time of cultivars was tightly associated with growth habits (Additional file 1: Table S2).

### Distribution frequency of *Vrn-1* alleles in varieties

Because no polymorphisms were found in the *Vrn-A1* and *Vrn-B3* alleles, we focused on *Vrn-B1* and *Vrn-D1*. The distribution frequency order of the dominant alleles was *Vrn-D1a* (23.70%) > *Vrn-D1b* (8.10%) *> Vrn-B1a* (2.50%) > *Vrn-B1b* (2.00%) (Table 3). Only one accession was found to carry *Vrn-B1b + Vrn-D1a*, and 125 accessions presented no dominant alleles. We also used the “consistency index” to evaluate the reliability between the allelic detection and speculated results as described by Stelmakh [19]. According to Stelmakh’s report, the accessions that contained at least one dominant allele were classified as spring types, whereas they were classified as winter types if they had three recessive alleles. Then, we found that nine accessions harbouring dominant *Vrn-B1a* or *Vrn-B1b* alleles as well as one accession harbouring *Vrn-B1b* + *Vrn-D1a* exhibited the highest rate of consistency (100%). Therefore, these accessions were classified as spring types, which were identical to the results of identification of growth habit in this research. The genotype rate of *vrn-B1* + *vrn-D1* (63.1%) dominated in all tested panels, and its consistency (96.8% or 95.2%) was also higher than that of *Vrn-D1a* (48.9%) and *Vrn-D1b* (25.0%). Therefore, *Vrn-D1*, especially *Vrn-D1b*, could not accurately estimate the growth habit.

**Table 3:** Distribution rates of *Vrn-1* and their consistency with results of identification of W/S growth habit

Genotype	Material number (only)	Frequency (%)	Speculation of winter/spring habit	Winter	Spring	Consistency (%)
*Vrn-B1a* (only)	5	2.5	Spring	0	5	100.0
*Vrn-B1b* (only)	4	2.0	Spring	0	4	100.0
*Vrn-D1a* (only)	47	23.7	Spring	24	23	48.9
*Vrn-D1b* (only)	16	8.1	Spring	12	4	25.0
*Vrn-B1b* + *Vrn-D1a*	1	0.5	Spring	0	1	100.0
*vrn-B1* + *vrn-D1*	125	63.1	Winter	119/117	4/6	96.8/95.2
Total	198	100.0		155/153	41/43	79.8/78.2

The values in parentheses represent the percentage in each group; “only” in parentheses indicates that the value is specific to the single genotype

### Effects of *Vrn-1* combinations on HD and FD

The effects of *Vrn-B1* + *Vrn-D1* combinations concerning HD and FD were examined. In total, there were 6 different types of genotypes grouped by combinations. Among them, 125 accessions had double-recessive *vrn-B1* + *vrn-D1* alleles. However, *Vrn-B1a + vrn-D1*, *Vrn-B1b + vrn-D1*, *vrn-B1 + Vrn-D1a*, *vrn-B1 + Vrn-D1b* alleles were carried by 5, 4, 47 and 16 varieties, respectively. Only 1 accession harboured double-dominant *Vrn-B1b + Vrn-D1a* alleles. Least significant range (LSR, a method of multiple comparison) tests revealed significant differences among the six groups with respect to the mean values of HD and FD in almost all environments (*P* < 0.05), with the exception that LSR tests for FD_15_ZMD were not significant (highlightedwith yellow). However, no significant differences in the mean values of each group across environments were revealed by the joint ANOVA results (highlighted with green) (Table 4).

**Table 4:** LSR method of multiple comparison for the effects of *Vrn-B1* combined with *Vrn-D1*

Types	N	HD_14_SQ	HD_15_SQ	HD_14_ZMD	HD_15_ZMD	HD_14_ZZ	HD_15_ZZ	HD_average
*vrn-B1 + vrn-D1*	125	183.1 ± 2.3(a)	188.5 ± 2.2(a)	172.2 ± 1.6(a)	159.1 ± 1.3(a)	181.8 ± 1.3(a)	188.7 ± 2.5(a)	178.9 ± 10.7(a)
*Vrn-B1a + vrn-D1*	5	179.1 ± 0.8(bc)	184.6 ± 0.8(bc)	171.2 ± 0.4(ab)	157.8 ± 0.5(ab)	178.6 ± 0.8(bc)	184.4 ± 1.6(bc)	175.9 ± 9.5(a)
*Vrn-B1b + vrn-D1*	4	177.5 ± 1.9(c)	182.5 ± 0.7(c)	170.0 ± 1.1(b)	156.7 ± 2.0(b)	177.3 ± 1.1(c)	181.7 ± 1.6(c)	174.3 ± 9.1(a)
*vrn-B1 + Vrn-D1a*	47	181.2 ± 3.2(ab)	186.5 ± 2.6(ab)	171.5 ± 1.3(ab)	158.8 ± 1.4(a)	179.8 ± 1.8(ab)	186.1 ± 2.6(ab)	177.4 ± 10.0(a)
*Vrn-B1b + Vrn-D1a*	1	180.5 ± NA(abc)	184.0 ± NA(bc)	171.0 ± NA(ab)	159.0 ± NA(a)	178.5 ± NA(bc)	184.5 ± NA(bc)	176.3 ± 9.8(a)
*vrn-B1 + Vrn-D1b*	16	180.8 ± 2.5(abc)	186.5 ± 2.7(ab)	171.5 ± 1.4(ab)	158.7 ± 1.1(ab)	179.7 ± 1.9(abc)	186.75 ± 2.9(ab)	177.4 ± 10.8(a)
Types	N	FD_14_SQ	FD_15_SQ	FD_14_ZMD	FD_15_ZMD	FD_14_ZZ	FD_15_ZZ	FD_average
*vrn-B1 + vrn-D1*	125	192.9 ± 1.9(a)	197.4 ± 1.0(a)	180.4 ± 1.9(a)	167.4 ± 1.7(a)	190.4 ± 1.5(a)	198.9 ± 1.8(a)	187.9 ± 11.1(a)
*Vrn-B1a + vrn-D1*	5	190.4 ± 0.8(ab)	195.0 ± 0.6(bc)	181.2 ± 1.1(a)	165.8 ± 1.0(a)	187.0 ± 0.9(bc)	195.0 ± 1.5(bc)	185.7 ± 10.3(a)
*Vrn-B1b + vrn-D1*	4	187.1 ± 0.9(b)	193.5 ± 0.7(c)	177.1 ± 1.2(b)	165.3 ± 1.3(a)	184.7 ± 1.5(c)	191.3 ± 2.2(d)	183.2 ± 9.8(a)
*vrn-B1 + Vrn-D1a*	47	191.0 ± 3.23(a)	195.9 ± 1.6(ab)	178.7 ± 1.9(ab)	167.2 ± 1.5(a)	187.9 ± 2.1(ab)	196.2 ± 3.0(abc)	186.2 ± 10.7(a)
*Vrn-B1b + Vrn-D1a*	1	190.0 ± NA(ab)	195.0 ± NA(bc)	181.0 ± NA(a)	167.5 ± NA(a)	187.0 ± NA(bc)	193.0 ± NA(cd)	185.6 ± 10.1(a)
*vrn-B1 + Vrn-D1b*	16	191.6 ± 3.3(a)	196.1 ± 1.6(ab)	178.6 ± 2.1(ab)	167 ± 1.1(a)	187.7 ± 2.5(ab)	196.7 ± 2.9(ab)	186.4 ± 11.5(a)

Letters in parentheses indicate a significant difference at the 0.05 level; decimal values preceded by “±” indicate standard deviation

Because the low frequency of the *Vrn-B1b + Vrn-D1a* type (0.5%) made it difficult to exactly compare this allelic combination with other genotypes, we focused on the other five combinations. With respect to their effects, we found that accessions with the *vrn-B1* + *vrn-D1* genotype presented the latest HD and FD (178.9 d), while varieties that harboured the *Vrn-B1b + vrn-D1* allelic combination presented the earliest HD and FD (174.3 d) as well as the shortest growth habit. This method could be useful for precisely identifying the differences in growth habits in each group individually. Then, analyses of *Vrn-1* combinations revealed that the effects of the dominant *Vrn-B1* genotype on HD and FD were stronger than those of the dominant *Vrn-D1* genotype. Finally, we concluded that the rank order of the effects on the growth period was as follows: *Vrn-B1b* > *Vrn-B1a > Vrn-D1b* > *Vrn-D1a* > *vrn-D1* = *vrn-B1* (Table 4).

### Allelic variations of *Ppd-*1 alleles

No polymorphisms were found in the promoter of *Ppd-A1* or *Ppd-B1*; thus, we focused on their internal variants. Here, variations in the junction sequences of *Ppd-B1* were investigated to analyse their allelic variations and effects, which were considered copy number variations (CNVs) [28]. For *Ppd-B1*, the *Ppd-B1a* gene has three types in terms of CNV, accounting for 33.8% (Truncated CS type), 8.6% (Intact CS type) and 35.3% (Sonora 64 type) (Table 5). According to a previous report, the first two genotypes were named *Ppd-B1c* and the Sonora 64 type was named *Ppd-B1a* [30]. For variations within *Ppd-B1*, 6 types of genotypic combinations were all detected because of their different types of combination. As to percentage, the “S: N: N” type constituted the largest proportion (34.34%, “Sonora 64 type” only), while the percentages of “N: I: N” (1.01%, “Intact CS type” only) and “S: N: T” (1.01%, “Sonora 64 type + Truncated CS type” for short) were the lowest (Additional file 1: Table S2; Table 5).

**Table 5:** Allelic variants of *Ppd-B1*

Genotype	Sonora 64 type	Intact CS type	Truncated CS type	*N*	Proportion (%)
*Ppd-B1*	No	No	No	61	30.81
	Yes	No	No	68	34.34
	No	Yes	No	2	1.01
	No	No	Yes	50	25.25
	Yes	No	Yes	2	1.01
	No	Yes	Yes	15	7.58
Total	35.3%	8.6%	33.8%	198	100

For *Ppd-D1*, the haplotypes identified among the materials were divided into three types in accordance with the reports of Guo et al. (2010) [31] and Chen et al. (2013) [13]: *Hapl I* (34.85%, sensitive), *Hapl II* (0.5%, Chinese Spring, insensitive), and *Hapl VII* (64.6%, sensitive). Only one variety (Chinese Spring) had a 2.0-kb deletion in the promoter region and thus should be designated *Ppd-D1a* (insensitive, theoretical relatively short HD and FD). Thus, *Hapl I* and *Hapl VII* could be considered recessive alleles in this study (Table 6).

**Table 6:** Allelic variants of *Ppd-D1*

Genotype	2-kb deletion	Transposable element (TE)insertion	5-bp deletion	16-bp insertion	Number	Proportion (%)
*Ppd-D1*	*Hapl I*				69	34.85
	*Hapl II*				1	0.51
	*Hapl VII*				128	64.65
Total					198	100

The method for dissecting the haplotypes of *Ppd-D1*was described by Guo et al. (2010)

### Effects of single *Ppd-1* alleles on HD and FD

One hundred and twenty-five materials that had double-recessive *Vrn* alleles (*vrn-B1* + *vrn-D1*) were selected to evaluate the influence of *Ppd-B1* or *Ppd-D1* on plant traits. There were four genotypes, but no significant differences were found among groups according to ANOVA results. Because inconsistencies between the ANOVA and LSR test results were sometimes detected, multiple comparisons were subsequently performed. The average values for phenotypes among the four groups differed significantly for only five traits (three traits in Zhumadian). Furthermore, varieties that harboured “Sonora 64 type” (“N: N: S”) showed the shortest HD and FD. This result was consistent with those reported by Díaz et al. (2012) [28] (Table 7).

**Table 7:** The effects of single *Ppd-B1* and *Ppd-D1*alleles on HD and FD

Type	*N*	HD_14_SQ	HD_15_SQ	HD_14_ZMD	HD_15_ZMD	HD_14_ZZ	HD_15_ZZ	HD_average
*N:N:N*	39	183.2 ± 2.7(ab)	188.7 ± 2.6(a)	172.1 ± 1.5(b)	159.2 ± 1.4(b)	181.8 ± 1.5(ab)	189.2 ± 2.6(a)	179.1 ± 10.9(a)
*T:N:N*	30	183.3 ± 2.1(ab)	188.6 ± 1.9(a)	172.7 ± 1.7(ab)	159.2 ± 1.2(b)	181.9 ± 1.4(ab)	188.4 ± 2.6(a)	179.0 ± 10.6(a)
*T:I:N*	10	184.1 ± 2.1(a)	189.3 ± 1.9(a)	173.6 ± 1.3(a)	160.2 ± 0.8(a)	182.7 ± 0.7(a)	189.7 ± 1.3(a)	179.9 ± 10.5(a)
*N:N:S*	44	182.6 ± 2.1(b)	188.2 ± 2.1(a)	171.8 ± 1.4(b)	158.8 ± 1.3(b)	181.7 ± 1.3(b)	188.2 ± 2.5(a)	178.6 ± 10.6(a)
Type	*N*	FD_14_SQ	FD_15_SQ	FD_14_ZMD	FD_15_ZMD	FD_14_ZZ	FD_15_ZZ	FD_average
*N:N:N*	39	193.3 ± 2.5(a)	197.3 ± 1.3(a)	180.4 ± 2.1(a)	167.5 ± 1.9(ab)	190.5 ± 1.8(a)	199.2 ± 2.1(a)	188.1 ± 11.3(a)
*T:N:N*	30	192.7 ± 1.6(a)	197.5 ± 0.9(a)	180.5 ± 1.9(a)	167.5 ± 1.4(b)	190.2 ± 1.4(a)	198.7 ± 2.2(a)	187.9 ± 11.1(a)
*T:I:N*	10	193.1 ± 0.9(a)	197.7 ± 0.8(a)	181.0 ± 1.4(a)	168.5 ± 1.4(a)	190.9 ± 0.8(a)	199.4 ± 0.8(a)	188.5 ± 10.8(a)
*N:N:S*	44	192.7 ± 1.5(a)	197.3 ± 0.8(a)	180.3 ± 1.8(a)	166.9 ± 1.6(b)	190.3 ± 1.4(a)	198.7 ± 1.5(a)	187.7 ± 11.2(a)
Type	*N*	HD_14_SQ	HD_15_SQ	HD_14_ZMD	HD_15_ZMD	HD_14_ZZ	HD_15_ZZ	HD_average
*Hapl I*	45	183.1 ± 2.2(a)	188.5 ± 2.0(a)	172.3 ± 1.5(a)	159.1 ± 1.1(a)	181.9 ± 1.3(a)	188.8 ± 2.2(a)	179.0 ± 10.6(a)
*Hapl VII*	77	183.1 ± 2.4(a)	188.5 ± 2.3(a)	172.2 ± 1.6(a)	159.1 ± 1.4(a)	181.8 ± 1.4(a)	188.6 ± 2.6(a)	178.9 ± 10.7(a)
Type	*N*	FD_14_SQ	FD_15_SQ	FD_14_ZMD	FD_15_ZMD	FD_14_ZZ	FD_15_ZZ	FD_average
*Hapl I*	45	192.7 ± 1.5(a)	197.3 ± 1.0(a)	180.7 ± 1.7(a)	167.2 ± 1.4(a)	190.4 ± 1.4(a)	199.1 ± 1.7(a)	187.9 ± 11.1(a)
*Hapl VII*	77	193.1 ± 2.1(a)	197.4 ± 1.1(a)	180.3 ± 2.0(a)	167.4 ± 1.8(a)	190.4 ± 1.5(a)	198.8 ± 1.9(a)	187.9 ± 11.2(a)

“*N*” in the “Type” column indicates that no target bands were amplified in the “Truncated CS type (425 bp)”, “Intact CS type (994 bp)"or”Sonora64 (223 bp)"CNVs at the *Ppd-B1* locus

Regarding *Ppd-D1*, two genotypes were found, and no significant differences were detected via the LSR method between groups. We predicted that two recessive types of alleles did not contribute to the advancement of heading and flowering time. With respect to the comparisons of the mean values of *Ppd-B1* and *Ppd-D1* on HD and FD, the effects of *Ppd-B1* were somewhat stronger than those of *Ppd-D1* (Table 7).

### Interactive effects of *Ppd-1* combinations on HD and FD

We also examined and assessed the interactive effects of *Ppd-B1* and *Ppd-D1* combinations. Similarly, no significant differences were found according to the ANOVA results. A total of eight genotypes that contained two *Ppd* alleles were surveyed, in which the type “N: N: S + *Hapl VII*” constituted the largest proportion (29, 23.6%), while the percentages of “T: I: N + *Hapl I* or *Hapl VII*” were the lowest (Table 8). With respect to their effects, the LSR method revealed significant differences in the mean values among groups for four traits (three in Zhumadian and one in Zhengzhou). We suspected that *Ppd-1*, especially *Ppd-B1*, functioned only in specific environments. Generally, the mean values of HD and FD were advanced by the *Ppd-1* combinations by only 0.2–0.5 d. Therefore, the effects of *Ppd-1* combinations were stronger than those of single *Ppd-B1* or *Ppd-D1* alleles but were far weaker than those of *Vrn* alleles (5–7 d earlier heading or flowering in Zhengzhou and Zhumadian). With respect to the individual genotypes, the rank order of their effects on growth period was as follows: *Ppd-1* > *Ppd-B1 > Ppd-D1*.

**Table 8:** Interactive effects of *Ppd-1* combinations on growth period and growth habit

Truncated CS type	Intact CS type	Sonara64 type	*Ppd-D1*	*N*	HD_14_SQ	HD_15_SQ	HD_14_ZMD	HD_15_ZMD	HD_14_ZZ	HD_15_ZZ	HD_average
No	No	No	*Hapl-I*	13	182.9 ± 2.4(a)	188.3 ± 2.6(a)	172.1 ± 1.3(bc)	159.0 ± 1.1(b)	181.5 ± 1.2(b)	188.4 ± 1.9(a)	178.7 ± 10.6(a)
Yes	No	No	*Hapl-I*	11	183.2 ± 2.2(a)	188.2 ± 1.6(a)	172.8 ± 1.9(abc)	159.1 ± 1.2(b)	181.8 ± 1.4(b)	188.8 ± 2.3(a)	179.0 ± 10.6(a)
Yes	Yes	No	*Hapl-I*	5	184.1 ± 1.7(a)	189.2 ± 2.2(a)	173.6 ± 1.3(a)	159.9 ± 0.4(ab)	183.1 ± 0.2(a)	190.1 ± 0.6(a)	180.0 ± 10.7(a)
No	No	Yes	*Hapl-I*	15	182.7 ± 2.2(a)	188.8 ± 1.8(a)	171.9 ± 1.1(c)	159.0 ± 1.2(b)	182.1 ± 1.3(ab)	188.7 ± 2.8(a)	178.9 ± 10.8(a)
No	No	No	*Hapl-VII*	26	183.5 ± 2.9(a)	188.9 ± 2.6(a)	172.1 ± 1.6(abc)	159.3 ± 1.6(ab)	182.1 ± 1.6(ab)	189.6 ± 2.8(a)	179.3 ± 11.0(a)
Yes	No	No	*Hapl-VII*	19	183.4 ± 2.0(a)	188.8 ± 2.1(a)	172.6 ± 1.6(abc)	159.2 ± 1.1(b)	181.9 ± 1.3(ab)	188.2 ± 2.8(a)	179.1 ± 10.7(a)
Yes	Yes	No	*Hapl-VII*	5	184.0 ± 2.7(a)	189.5 ± 1.8(a)	173.6 ± 1.5(ab)	160.5 ± 1.1(a)	182.3 ± 0.9(ab)	189.4 ± 1.8(a)	179.9 ± 10.5(a)
No	No	Yes	*Hapl-VII*	29	182.5 ± 2.1(a)	187.8 ± 2.2(a)	171.8 ± 1.6(c)	158.7 ± 1.4(b)	181.5 ± 1.3(b)	187.9 ± 2.3(a)	178.4 ± 10.5(a)
Truncated	Intact	Sonara64	*Ppd-D1*	N	FD_14_SQ	FD_15_SQ	FD_14_ZMD	FD_15_ZMD	FD_14_ZZ	FD_15_ZZ	FD_average
CS type	CS type	type									
No	No	No	*Hapl-I*	13	192.8 ± 1.8(a)	197.1 ± 1.3(a)	180.6 ± 1.7(a)	167.3 ± 1.2(b)	190.41 ± 1.3(a)	198.9 ± 2.0(a)	187.9 ± 11.1(a)
Yes	No	No	*Hapl-I*	11	192.4 ± 1.1(a)	197.3 ± 0.8(a)	181.1 ± 2.1(a)	167.3 ± 1.4(b)	190.0 ± 1.2(a)	199.0 ± 1.8(a)	187.9 ± 11.0(a)
Yes	Yes	No	*Hapl-I*	5	193.4 ± 0.5(a)	197.9 ± 1.1(a)	181.2 ± 1.4(a)	167.8 ± 1.3(ab)	191.1 ± 0.7(a)	199.8 ± 0.4(a)	188.5 ± 11.2(a)
No	No	Yes	*Hapl-I*	15	192.6 ± 1.8(a)	197.5 ± 0.8(a)	180.4 ± 1.9(a)	167.0 ± 1.7(b)	190.6 ± 1.7(a)	198.8 ± 1.7(a)	187.8 ± 11.3(a)
No	No	No	*Hapl-VII*	26	193.6 ± 2.9(a)	197.5 ± 1.4(a)	180.3 ± 2.4(a)	167.7 ± 2.2(b)	190.5 ± 2.0(a)	199.3 ± 2.1(a)	188.2 ± 11.4(a)
Yes	No	No	*Hapl-VII*	19	192.9 ± 1.9(a)	197.6 ± 0.9(a)	180.1 ± 1.8(a)	167.6 ± 1.5(b)	190.4 ± 1.5(a)	198.6 ± 2.4(a)	187.9 ± 11.2(a)
Yes	Yes	No	*Hapl-VII*	5	192.8 ± 1.3(a)	197.5 ± 0.5(a)	180.8 ± 1.4(a)	169.3 ± 1.1(a)	190.8 ± 1.1(a)	199.1 ± 1.1(a)	188.4 ± 10.6(a)
No	No	Yes	*Hapl-VII*	29	192.8 ± 1.4(a)	197.2 ± 0.8(a)	180.2 ± 1.8(a)	166.8 ± 1.6(b)	190.2 ± 1.2(a)	198.6 ± 1.4(a)	187.7 ± 11.2(a)

Letters in parentheses indicate a significant difference at the 0.05 level; decimal values preceded by “±” indicate standard deviation; ‘*N*’ indicates no target bands were amplified in ‘Truncated CS type’, ‘Intact CS type’ and ‘Sonara64’ of CNVs at *Ppd-B1* locus

## Discussion

### Consistency of marker analysis and growth habit identification

Wheat is the major crop in the YHW in terms of yield and area in China. This region is located in the transition zone of winter and spring wheat cultivation, where semi-winter varieties and weak spring cultivars are also planted. However, inconsistencies sometimes occur between registered and empirical results. Hence, the precise identification of winter/spring growth habits for newly registered varieties is necessary and helpful not only for the rational use of varieties but also for the provision of vital information for breeders in the YHW. Here, growth habits were examined during a two-year period via a novel field spring sowing identification method and materials along with marker-assisted selection (MAS).

We believe that our identification method in the field is more practical than that conducted in greenhouses, where the materials are grown under conditions closely related to those in the field. Furthermore, correlation analysis revealed that the phenotypic data during two years were also consistent between years. In comparison with registered information, 10 of 155 (6.45%, 2016) winter wheat varieties were inconsistent (Zhongchuang805, Taikong6, Xiaoyan22, etc.), and similar situations were observed in other groups—in particular, 43 spring wheat varieties containing 13 (25.6%, 2016) inconsistent samples (Bainong3217, Luohan6, Xinong979, etc.). We doubted that the reason for this situation was due to their early registration before the materials were rigorously identified. In total, the consistency was approximately 90%, although some varieties presented discrepancies, and our method was more convenient than the report of Gardener and Barnett [32].

More vital clues that we wanted to examine included the consistency between vernalization alleles and growth habit. The results indicated that all ten *Vrn-B1* genotypes of spring wheat varieties presented a value of 100%, whereas *Vrn-D1* exhibited lower results. Among the 43 cultivars ranked as grade 5 (data from2016, Xinzheng), 37 (86.04%) carried at least one of the tested dominant vernalization alleles and were classified as spring varieties; the other 6 varieties carried the recessive alleles at the three vernalization loci. For winter types, 131 of 155 (84.51%) accessions presented similar consistency. We predicted that there are two main factors that could be responsible for this phenomenon. First, a single individual is genotyped, whereas the phenotype is assessed on a plot scale of multiple individuals, and there may be some variation among individual seeds. Second, two other major pathways also control heading and flowering dates in plants, i.e., the phytohormone gibberellic acid (GA) and the autonomous pathways, in addition to the vernalization and photoperiod pathways [33, 34].

Allelic distributions of *Vrn-1* revealed trends and orientations in the YHW.

As no dominant allele of *Vrn-A1* or *Vrn-B3* was detected, which are probably the two genes that have the strongest effects of those examined, the allelic distributions of the dominant *Vrn-B1* and *Vrn-D1* alleles are likely responsible for the spring genotypes of wheat varieties. Indeed, the scarcity and decreasing frequency of the *Vrn-A1* and *Vrn-B3* loci in the YHW have been previously discussed by Zhang et al. (2008) and Chen et al. (2013) [4, 13]. We suspected that the frequencies of recessive *vrn-B1* and *vrn-D1* likely increased via direct selection due to their contributions to yield traits because of the long maturation period. This inference was in agreement with that reported in the literature [14, 35]. Furthermore, the frequency of dominant *Vrn-D1* was higher than that of *Vrn-B1* in our tested materials. These results were also consistent with the previous report of Zhang et al. (2015) [36].

Although only 10 dominant *Vrn-B1* alleles (5.0%) were discovered in the spring genotypes, the consistency of the marker-growth habit (100%) was better than that of *Vrn-D1*. Additionally, we found that accessions with single *Vrn-B1b* alleles exhibited the earliest HD and FD in the six environments; these effects were stronger than those for*Vrn-B1a*, *Vrn-D1b* and *Vrn-D1a*. These results were consistent with those of previous studies in which the rank order was *Vrn-A1 > Vrn-B1 > Vrn-D1* [37]. Santra et al. (2009) reported that a novel *Vrn-B1b* allele that resulted from a 36-bp deletion within intron 1, which is referred to as ‘Alpowa’ and carries the winter growth habit alleles *vrn-A1* and *vrn-B1*, was likely to cause a spring growth habit; however, the authors did not provide sufficient evidence [14].

The dominant *Vrn-D1* locus occurs in the most popular types and is distributed throughout nearly the entire wheat production region [4, 13]. The previous data also established that carriers of *Vrn-A1* or *Vrn-D1* tend to produce longer spikes than do carriers of*Vrn-B1*. As a result, the *Vrn-D1* genotypes were prevalent in China [38, 39]. In the present study, in terms of *Vrn-D1*, the allelic frequency reached 31.8%, which represented the most dominant allele distribution in the population. Thus, the proportion was similar to previous reports; however, poor consistency in the growth habit of Zhengzhou was observed. Thus, it would be interesting to test whether *Vrn-D1* and other alleles interact to influence growth habits and period (HD and FD).

The results of the *Vrn-1* combination analysis revealed that only one accession carried *Vrn-B1b* + *Vrn-D1a* alleles; thus, the samples were so limited that phenotypic data were not statistically representative. For genotypic analysis, in combination with growth habit identification, 63.1% of the materials that had three recessive alleles all belonged to winter or semi-winter wheat (grade 0–4). This tendency was in accordance with that reported by Sun et al. (2009, 61.1%). Moreover, the ANOVA and LSR tests revealed that the mean values of the HD, FD and growth habit among six genotypes differed significantly, which also indicated that the *Vrn-1* combinations were tightly associated with phenotypes. Although the effects of these combinations on HD and FD were not definitively stronger than those of single *Vrn-B1* alleles, the growth habit level was divided in greater detail. This division will enable a more precise identification of vernalization requirements for accessions at molecular levels.

### Interactive effects detected between the *Ppd-B1* and *Ppd-D1* alleles

Photoperiod responses are controlled by members of the pseudo-response regulator (*PRR*) gene family in plants. In general, the potential of *Ppd-1* alleles to affect insensitivity has been ranked as *Ppd-D1* > *Ppd-B1* > *Ppd-A1* [17, 38]. However, in the present study, only one accession (Chinese spring) was found to carry a 415-bp band that indicated a genotype of *Ppd-D1a*. Thus, it was difficult to precisely evaluate its effect on phenotype statistically. According to the criterion of previous research, two sensitive haplotypes (*Hapl I* and *Hapl II*) of *Ppd-D1* were used for the evaluation for their distribution and effects [13, 31].

For *Ppd-B1*, we only examined the polymorphisms of CNVs of the *Ppd-B1* locus because of its tight association with heading and flowering time. Indeed, Zhang et al. (2015) designated eight haplotypes according to the combinations of CNVs of *Ppd-B1* and found that the cultivar with *Ppd-B1*_*hapl-VI* demonstrated the earliest heading and flowering times [36]. However, the results were not consistent with those of Díaz et al. (2012) [28]. In the present study, 125 accessions carrying recessive *vrn-B1* and *vrn-D1* alleles were selected. With respect to *Ppd-B1*, we found that wheat cultivars with “Sonora 64” *Ppd-B1a* alleles flower earlier than those with “Chinese Spring” alleles, which was in accordance with Díaz et al. (2010). Furthermore, we found that six types of combinations emerged, and the “Truncated CS type” and “Intact CS type” did not simultaneously emerge for *Ppd-B1*. These results were also not the same as those reported by Chen et al. (2013). We suspected that the complex genetic background (genotypes mixed with *Vrn-1* genes) would hinder us from providing definitive results. Thus, we believed that our method was possibly more reliable than previous methods because of the uniform background [30]. With respect to *Ppd-D1a*, the rare diversity of the *Ppd-D1* allele could not be used to exactly evaluate the effects of the variants, and no significant differences were observed between the two haplotypes (*Hapl I* and *Hapl II*).

### Comparison of effects on growth period (HD and FD) between the *Vrn-1* and *Ppd-1* alleles

Moreover, from a comprehensive perspective, we concluded that, compared with *Ppd-1*, *Vrn-1* played a major role in regulating heading and flowering traits as well as growth habit. At the *Vrn-1* locus, cultivars with the *Vrn-B1b + vrn-D1* (174.3 d for HD, 183.2 d for FD) allele both headed and flowered earlier by approximately 4 days than did cultivars with the *vrn-B1 + vrn-D1* (178.9 d for HD, 187.9 d for FD) allele (Table 4). Whereas at the *Ppd-1* locus, cultivars with the “*N: N: S*” allele combination (178.6 d for HD, 187.7 d for FD) both headed and flowered approximately 1 day earlier than did cultivars with the “*T: I: N*” allele combination (179.9 d for HD, 188.5 d for FD) (Table 7). Indeed, the interactive effects of *Vrn-1* and *Ppd-1* gene combinations were also detected in our research. However, the results of ANOVA and LSR tests revealed weak interactions between *Vrn-B1* and *Ppd-B1*, *Vrn-D1* and *Ppd-D1*, *Vrn-B1*and *Ppd-D1*, *Vrn-D1*and *Ppd-B1* (data not shown). We suspected that the complex genetic background in natural populations would make it difficult to reveal this interaction. In a previous study, Shcherban et al. (2014) also found that the haplotypes *Ppd-D1a*/*Vrn-B1a* or *Ppd-D1a*/*Vrn-B1a* did not differ significantly in heading time from the respective *Vrn-1*haplotypesharbouring the sensitive allele *Ppd-D1b* [40]. This finding suggests that it is better for us to examine the interaction between *Ppd-1* and *Vrn-1* in biparental populations.

## Conclusion

In the present study, we dissected the *Vrn-1* and *Ppd-1* gene composition and found that *Vrn-1*, rather than *Ppd-1*, played a major role in controlling vernalization and photoperiod responses in this region. The work will be helpful for guiding the breeding of wheat in the Yellow and Huai wheat production region.

## Methods

We tested 198 cultivars (lines) including historic varieties, commercial varieties, and newly bred varieties originating from the YHW. Among them, 159 accessions were from Henan, 10 accessions were from Shandong, 10 accessions were from Shaanxi, 8 accessions were from Jiangsu,4 accessions were from Hebei, 4 accessions were from Beijing, 1 accession was from Shanxi, 1 accession was from Anhui, and 1 accession (Chinese Spring) was from Sichuan (Table 1). The entire original source of the plant materials used in our study was kindly provided by other labs. We complied with the Convention on the Trade in Endangered Species of Wild Fauna and Flora: https://www.cites.org.

### Characterization of winter/spring growth habits

Although the growth habit for assessing vernalization is already well established, identification of the exact materials involved is necessary because of differences in environmental conditions. The tested materials were planted at the Zhengzhou Scientific Research and Education Center of Henan Agriculture University (113.7°E, 34.7°N) on 12 March 2015 and at another test site [ZhengHan Seed Technology Co. Ltd., XinZheng (113.7°E, 34.4°N)] on 12 March 2016. Seeds were sown in 1.0-m rows, and individual seeds were spaced 6.67 cm apart; 15 seedlings were reserved per row after wheat seedling emergence. Two replications were planted for reliable data collection. The stage of maturity and percentage of headed spikes were recorded on 25 June in the same year; we repeated these measurements one week later. The growth habit of the materials was divided into grades numbered 0 to 5. The criteria were as follows: 0, no jointing and booting; 1, partial main stem headed; 2, main stem and a few tillers headed; 3, normal heading but abnormal grain filling and immature; 4, normal heading and grain filling but premature; 5, normal maturity.

### Identification of HD and FD

The varieties used to assess agronomic traits were planted on 9 October 2013 and 2014 in Zhengzhou (113.7°E, 34.7°N), on 15 October 2013 and 17 October 2014 in Shangqiu (115.7°E, 34.5°N), and on 19 October 2013 and 5 November 2014 in Zhumadian (114.0°E, 32.9°N). All of these locations differed significantly in day length and climatic factors in Henan Province. Each material was planted in two 1.5-m rows; there were 110 seeds per row, and the rows were spaced 23 cm apart. Two replications were planted at each location. Field management practices during our experiments were in accordance with agronomic practices commonly used in the area. The HD and FD were assessed on a plot scale of multiple individuals when more than half of the individual seedlings exhibited classic morphological traits for these events.

### DNA extraction and diagnostic markers for *Vrn-1* and *Ppd-1*

DNA was extracted from the seedlings in accordance with a modified SDS-phenol-chloroform method [27]. The primers used were based on those described in many previous reports and were synthesized by Sangon Biotech Co., Ltd. (Shanghai) (Additional file 2: Table S1). To recognize the segments amplified from *Ppd-B1*, accessions harbouring 994 bp, 425 bp, and 223 bp were designated intact Chinese Spring type (I), truncated Chinese Spring type (T), and Sonora 64 type (S), respectively. If no bands were amplified in the materials, the genotypes were referred to as null (N) [28].

### PCR amplification and electrophoresis

PCR amplification reactions were conducted in a 12-μL reaction flask containing 40 ng of genomic DNA, each primer at 2.5 μM, each dNTP at 200 μM, 1× buffer containing1.5 μM MgCl_2_, and 0.5 units of *Taq* polymerase. We used a Bio-Rad thermocycler with the following PCR conditions: 94 °C for 3 min; 34 cycles of 94 °C for 30 s, 50 °C to 65 °C for 30 s (annealing temperatures for each primer pair are listed in Additional file 2: Table S1), and 72 °C for 1 min; and a final 10-min extension at 72 °C for preservation. The PCR products were separated by electrophoresis either on a 0.8–1.2% agarose gel stained with ethidium bromide (EB) or an 8% nondenaturing polyacrylamide gel and visualized with silver staining [29].

### Statistical analysis

A consistency index (%) was used; this index indicated the number of accessions that originated from the consistent results of both genotype and field identifications divided by the total number of materials. The phenotypic data were imported into R software (R 3.4.1) for analysis via ANOVA, Student’s *t*-tests and correlation; we used the “reshape” and “agricolae” packages to perform these analyses and the “ggplot2” package for graphical construction.

## Additional file

Additional file 1: **Table S2.** The genotypes of *Ppd-1* and their effects on heading and flowering dates. (XLS 25 kb)

Additional file 2: **Table S1.** Primers used in this study. (XLS 71 kb)

## Abbreviations

YHW: Yellow and Huai wheat production region.
HD: Heading date.
FD: Flowering date.
ZZ: Zhengzhou.
ZMD: Zhumadian.
SQ: Shangqiu.
ANOVA: Analysis of variance.
LSR: Least significant range method of multiple comparison.
CNVs: Copy number variations.
MAS: Marker-assisted selection.
PRR: Pseudo-response regulator.
WS: Winter/Spring growth habit.
